# A machine learning framework for multi-hazards modeling and mapping in a mountainous area

**DOI:** 10.1038/s41598-020-69233-2

**Published:** 2020-07-22

**Authors:** Saleh Yousefi, Hamid Reza Pourghasemi, Sayed Naeim Emami, Soheila Pouyan, Saeedeh Eskandari, John P. Tiefenbacher

**Affiliations:** 1 Soil Conservation and Watershed Management Research Department, Chaharmahal and Bakhtiari Agricultural and Natural Resources Research and Education Center (AREEO), Shahrekord, Iran; 2 Department of Natural Resources and Environmental Engineering, College of Agriculture, Shiraz University, Shiraz, Iran; 3 Forest Research Division, Agricultural Research Education and Extension Organization (AREEO), Research Institute of Forests and Rangelands, Tehran, Iran; 4 Department of Geography, Texas State University, San Marcos, TX 78666 USA

**Keywords:** Environmental sciences, Natural hazards

## Abstract

This study sought to produce an accurate multi-hazard risk map for a mountainous region of Iran. The study area is in southwestern Iran. The region has experienced numerous extreme natural events in recent decades. This study models the probabilities of snow avalanches, landslides, wildfires, land subsidence, and floods using machine learning models that include support vector machine (SVM), boosted regression tree (BRT), and generalized linear model (GLM). Climatic, topographic, geological, social, and morphological factors were the main input variables used. The data were obtained from several sources. The accuracies of GLM, SVM, and functional discriminant analysis (FDA) models indicate that SVM is the most accurate for predicting landslides, land subsidence, and flood hazards in the study area. GLM is the best algorithm for wildfire mapping, and FDA is the most accurate model for predicting snow avalanche risk. The values of AUC (area under curve) for all five hazards using the best models are greater than 0.8, demonstrating that the model’s predictive abilities are acceptable. A machine learning approach can prove to be very useful tool for hazard management and disaster mitigation, particularly for multi-hazard modeling. The predictive maps produce valuable baselines for risk management in the study area, providing evidence to manage future human interaction with hazards.

## Introduction

Human interactions with natural extreme events, or hazards, are increasing globally^1^. Natural disasters have affected people and natural environments generating vast economic losses around the world. However, in some developed counties disasters have been decreasing since 1900^2,3^.

Hazard is the probability of occurrence in a specified period and within a given area of a potentially damaging of a given magnitude^4,5^. The definition incorporates the concepts of location (where?), time (when, or how frequently?) and magnitude (how large?). Total risk (R) means the expected number of lives lost, person injured, damage to property, or disruption of economic activity due to a particular natural phenomenon, and is therefore the product of specific risk (RS) and elements at risk (E)^6^. In addition, RS is the expected degree of loss due to a natural phenomenon.

Landscapes around the world are reflections of diverse natural processes. The probabilities of extreme natural events are typically greater in more natural areas and are, in fact, extensions of natural systems. Exposure of people to these extreme natural processes could be reduced and limited if predictive models based on new approaches and deeper knowledge of effective factors were employed^7^. Mountainous areas are commonly sites of snow avalanches^8,9^, landslides^4,10^, floods^11,12^, mudflows^13^, ice avalanches^14^, soil erosion^15–17^, rock falls^18^, and wildfires^19–24^.

Most studies focus on a single hazard, even when there are multiple hazardous processes affecting the same landscapes^8,25–30^. However, hazards sometimes interact with each other. Sometimes, the mitigation of one hazardous process may intensify another’s frequency, duration, distribution, or intensity^31^. Studying natural hazards separately, especially in mountainous regions, may produce miscalculations of risk (or the probability of occurrence of the specific extreme natural event) in those areas. Multi-hazard risk assessment (the collective likelihood of experiencing an extreme natural event among a set of hazards) could be useful for controlling the interactions of hazards^32^.

Snow avalanches, landslides, wildfires, land subsidence, and floods are the most important risks in many mountainous regions of the world^8,18,33,34^. These five hazards can impact and interrupt systems (transportation, electrical power, water provisioning systems, and others), processes (trade, travel, extraction, shipping), places (residential areas, commercial districts, industrial areas, recreational sites), and people in risk-prone areas^33,34^. Multi-hazard risk mapping is an important need for land use management at provincial and national scales^4,33–37^. Multi-hazard mapping is receiving increasing attention^1,7,38–45^. Multi-hazard analysis has been conducted in mountainous regions of Iceland, New Zealand, Iran, and Tajikistan. In Iceland, a general method was developed for analysis of snow avalanche, rock-fall, and debris-flow hazards^7^. Schmidt et al.^46^ developed an approach for multi-risk modeling in New Zealand, creating an adaptable software prototype that allows researchers to ‘plug in” natural processes of interest. Pourghasemi et al*.*^45^ undertook a multi-hazard risk assessment based on machine learning methods in Fars Province, Iran. They considered floods, forest fires, and landslides in their study area. In another study, multiple hazards (rockslides, ice avalanches, periglacial debris flows, and lake-outburst floods) were assessed in a mountainous region of Tajikistan to develop a comprehensive regional-scale map of hazards for the study area^47^.

Though these studies exemplify multi-hazard mapping, a comprehensive study on multi-hazard assessment by machine learning models is lacking for mountainous areas. The development of multi-hazard risk mapping approaches using new methods is critical for effective management of hazards in some regions. Iran, in fact, is a country that has an extensive array of hazards (flood, landslide, earthquake, drought, dust storm, soil erosion, snow avalanche, etc.) due to the diverse geomorphological and climatic zones^28,12,48–50^. Snow avalanches, landslides, subsidence, wildfires, and floods occur annually in the Zagros and Alborz mountain chains in Iran; people are more numerous in these regions than in other parts of the country^51–54^. In this study, five major natural events (flood, landslides, land subsidence, snow avalanches, and wildfires) in Chaharmahal and Bakhtiari Province provide the basis for a multi-hazard risk assessment map. The main objective is to evaluate machine learning models as useful, universal, and accurate multi-hazard mapping products that can be applied by land use managers and planners. Based on a review of the literature, we have selected a set of machine learning models including; generalized linear model (GLM)^55–57^, random forest (RF)^17,58,59^, a support vector machine (SVM)^60–62^, boosted regression trees (BRT)^63–65^, mixture discriminate analysis (MDA)^66,56^, multivariate adaptive regression splines (MARS)^67,56,68^, and functional discriminant analysis (FDA)^17,66^ for multi-hazard mapping. Finally, based on the accuracies of the models, available data, and the sources of models, the SVM, GLM and FDA algorithms were used to map hazards in the study area.

### Study area

**Figure 1:**
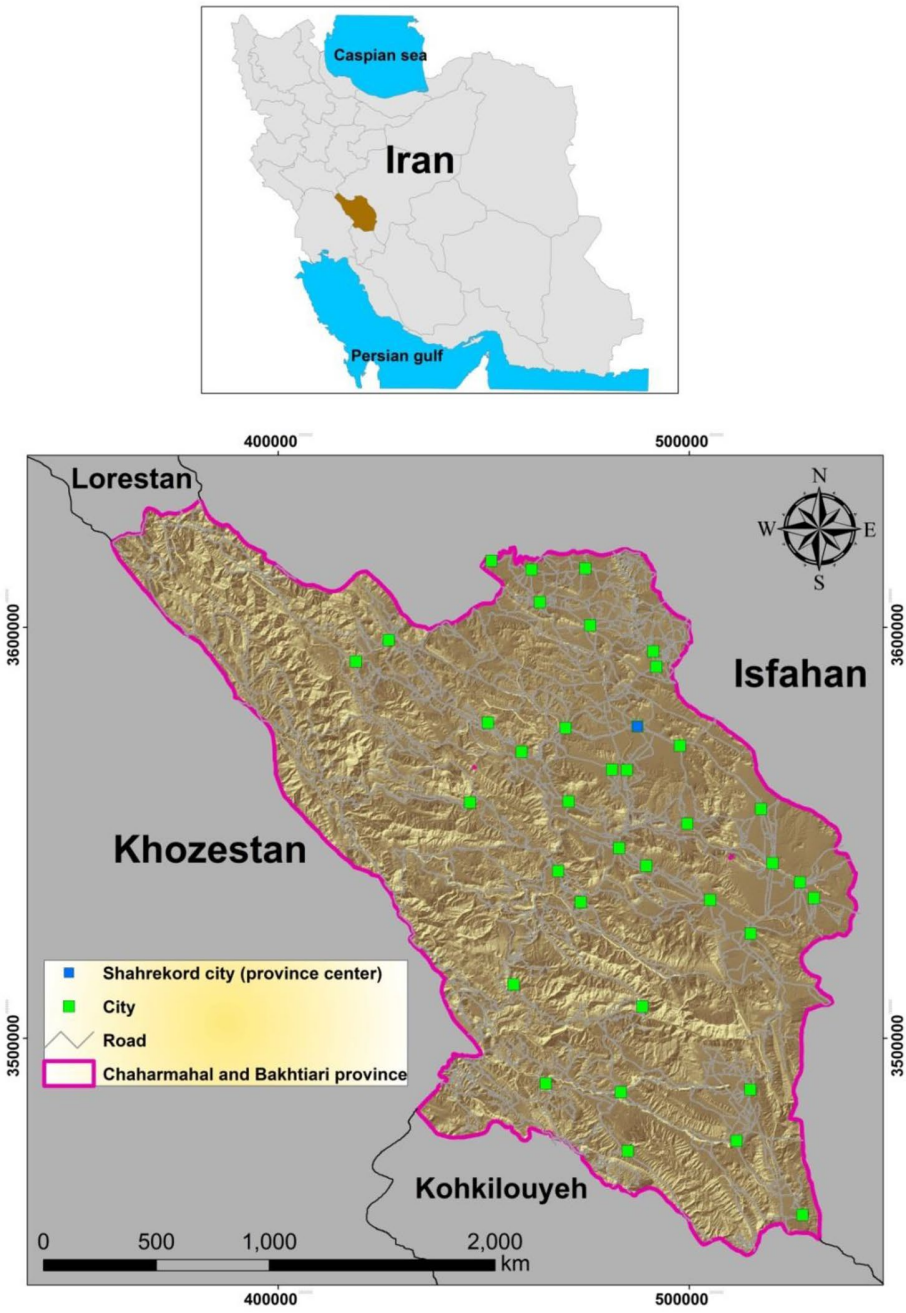
Location of the study area of Chaharmahal and Bakhtiari Province, Iran.

Chaharmahal and Bakhtiari Province is in southwestern Iran (Fig. 1). It is defined by a rectangle with sides at 31° 9′ and 32° 38′ N and 49°30′ and 51° 26′ E. The province contains ten counties (Ardal, Borojen, Shahrekurd, Farsan, Lordegan, Kiar, Khanmirza, Kohhrang, Ben and Saman) and covers an area of 16,553 km^2^ (1% of Iran). Elevation ranges from 778 m to 4,203 m above sea level and the mean elevation in the province is 2,153 m, making it the highest province in Iran. The population of the province is 947,000. Floods, mass movements, land subsidence, wildfires, and snow avalanches occur here regularly^69^.

### Methodology

**Figure 2:**
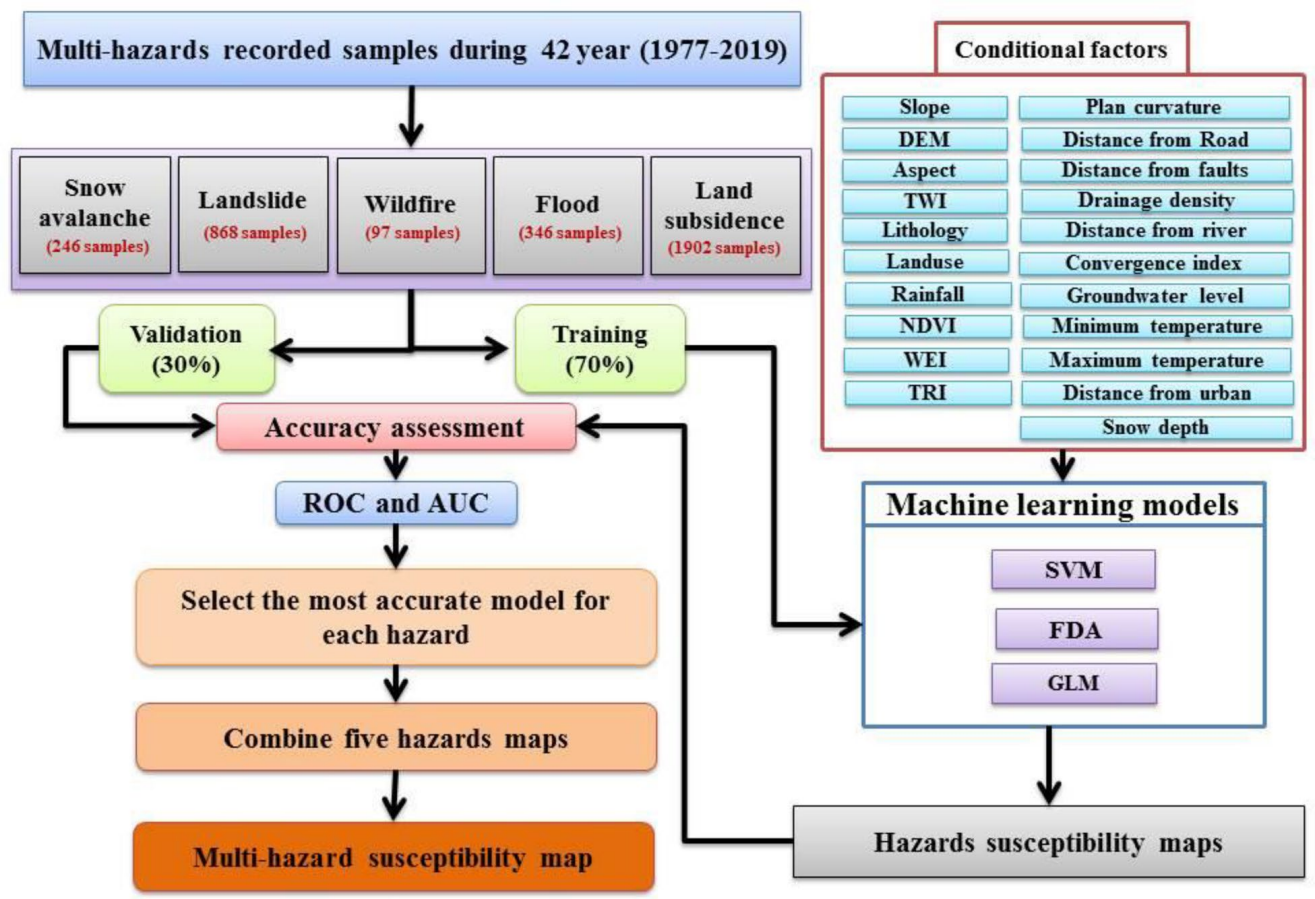
Flowchart of the study methodology.

This study involved three main activities (Fig. 2): (1) Collection of extreme event data through extensive field work and assessments of government reports over a 42-year period (1977–2019); (2) Identification of the most important effective factors for each hazard through a literature review; (3) Hazard modeling using a generalized linear model (GLM), a support vector machine (SVM) model, and a functional discriminant analysis (FDA) model and construction of multi-hazard risk maps (MHRM) using the models that were most accurate for each hazard.

### Hazards data inventory

**Figure 3:**
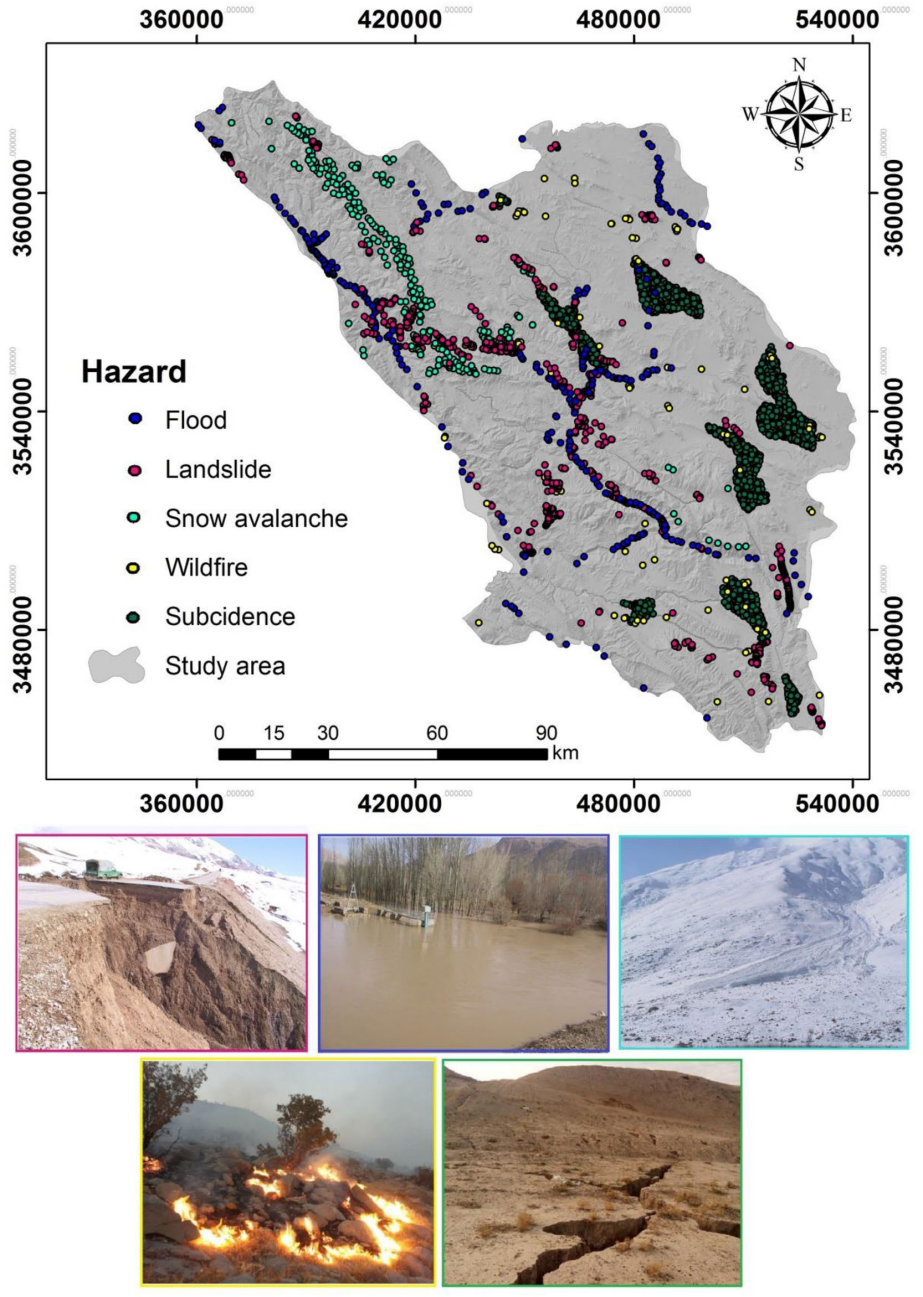
Distribution of the occurrence of the five hazards between 1977 and 2019 in Chaharmahal and Bakhtiari Province (**a**), and images of the five natural extreme events in the study area (**b**) taken by Saleh Yousefi (First author).

This study identified 3,455-point locations signifying the sites of five types of extreme hazardous events that occurred over a 42-year period in the Chaharmahal and Bakhtiari province through field surveys and examination of scientific reports (Fig. 3). These events included 246 snow avalanches, 97 wildfires, 346 floods, 868 landslides, and 1902 cases of land subsidence. The machine learning models in this study required data from both hazard and non-hazard locations to conduct modeling. Equal numbers of non-hazard locations were randomly sampled to balance the hazard locations^21–23,66,70^. The samples were divided into two groups for training (70%) and for validation (30%)^21,23,24^.

#### Data collection of the effective factors for five hazards

Based on both a review of previous studies and a compilation of experts’ suggestions, effective factors for each hazard were measured and mapped in raster layers of 10 × 10 m pixel size in ArcGIS 10.4.2. The effective factors (Table 1) fell into five categories: topography (DEM, slope, topographic wetness index, plan curvature, aspect, and convergence index), geology (lithology and distance from a fault), hydrology and climatology (precipitation, distance from a river, groundwater depth, drainage density, absolute minimum temperature, wind exposure index, absolute maximum temperature, and snow depth), society (distance from a road and distance from an urban areas), and vegetation/land cover (land use and NDVI). The topographic factors were extracted from 1:25,000 topographic maps obtained from the Iranian National Cartographic Center. The geological factors were acquired from a geologic map at a scale of 1:100,000, acquired from the Iranian Geology Organization. Hydrological and climatic factors were measured using data from 28 meteorological stations, digital stream layers, and 895 piezometric wells. These data were obtained from the Regional Water Company of Chaharmahal and Bakhtiari. The social factors were extracted from road networks and residential areas mapped on 1:25,000 topographic maps. The vegetation factors were discerned from Landsat 8 OLI images from June 2018. In addition, to evaluation of the importance of the effective factors for each hazard, specific factors were selected for modeling specific hazards: 12 for wildfires, 8 for snow avalanches, 12 for landslides, 12 for land subsidence, and 12 for floods.

**Table 1:** The effective factors for susceptibility mapping of five hazards.

Effective factors		Hazard				
Full name	Abbreviation	Flood (F)	Wildfire (WF)	Snow avalanche (SA)	Landslide (L)	Land subsidence (LSu)
Rainfall	R	*	*			
Digital elevation model	DEM	*	*	*	*	*
Land use	LU	*		*	*	*
Lithology	Lit	*			*	*
Fault distance	FD				*	*
Slope	S	*	*	*	*	*
River distance	RD	*	*		*	*
Groundwater level	GL					*
Normalized difference vegetation index	NDVI	*	*			*
Road distance	RoD	*	*		*	*
Topographic wetness index	TWI	*	*		*	*
Plan curvature	PC	*		*	*	*
Aspect	A	*	*	*	*	*
Drainage density	DD	*			*	
Convergence Index	CI				*	
Minimum temperature	MinT		*			
Maximum temperature	MaxT		*			
Urban area distance	UD		*			
Wind exposition index	WEI		*	*		
Terrain ruggedness index	TRI			*		
Snow depth	SD			*		

#### Application of machine learning models

Three state-of-the-art machine learning models were applied in present study to construct the hazard risk maps. Each is explained below.

##### Functional discriminant analysis (FDA)

FDA creates a statistical method to analyze effective factors. It can generally be said that models based on discrimination do unsupervised work so that each class is subdivided into its own subclass; each subclass is given a special value^71,72^. The FDA model is a special combination of regression models that implements a hidden process for each class in the modeling process, especially when conducting complex class modelin^73,74^. The FDA model is similar to other statistical methods, so it can perform just as well^75^. But, since the FDA model is nonparametric, it has been used in a wide range of fields^76^. The FDA model is new to analyses of data, but it has been convenient to use it as a replacement for functions. Therefore, more attention should be paid to this method^77^.

##### Generalized linear model (GLM)

The GLM is regression-based so it can reveal differences between variables^78^. The GLM is created from several linear models, and it constructs a best regression model that can predict multiple events^79–81^. Some researchers have reported that GLM is most often used for spatial modeling^55,82–85^. In general, the GLM uses multiple regression to increase accuracy and quality of the results because it can establish a very clear relationship between the dependent and independent variables^86^.

##### Support vector machine (SVM)

SVM uses both classification and regression, based on the concept of controlled learning. Results have shown that it generates the smallest clustering errors^87^. Since this model's approach is based on statistical learning theory, it reduces errors and identifies the optimal response^88^. SVM indicates performance estimation by answering a convex optimization problem^89,90^. The SVM model provides a very important advantage: it identifies and analyzes layers effectively^91^.

#### Multi-hazards risk mapping

**Figure 4:**
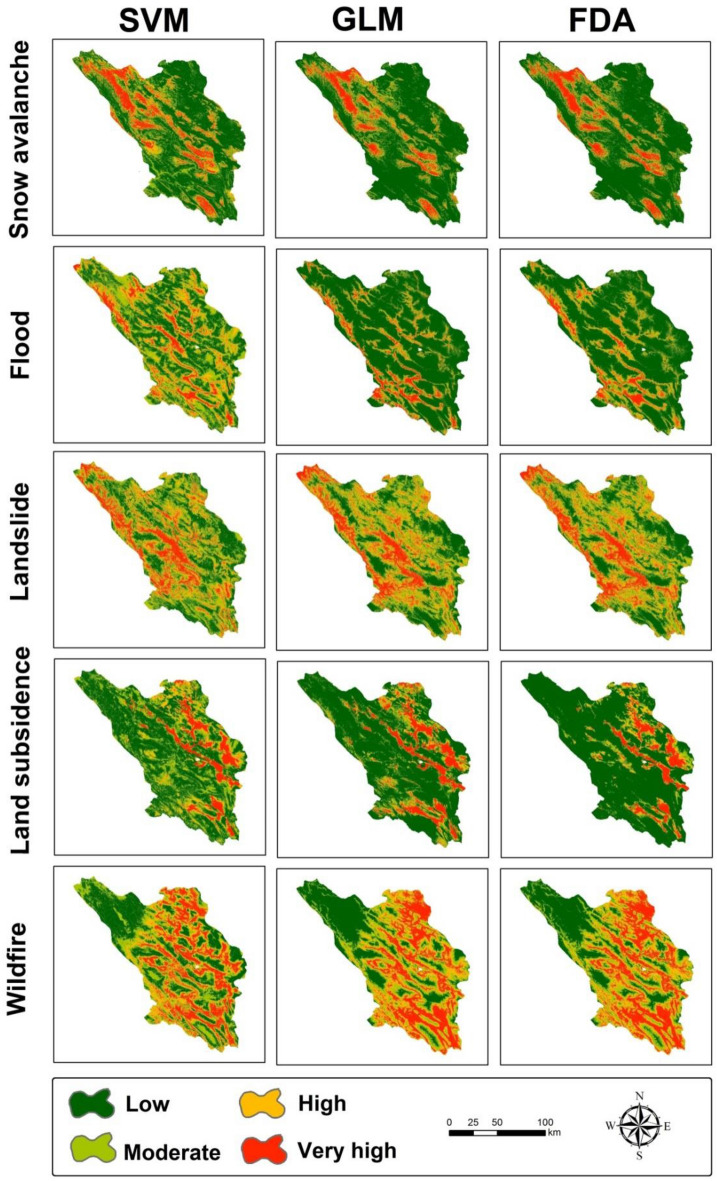
The risk maps of the five hazards created from the three machine learning models for Chahaharmahal and Bakhtiari Province.

**Figure 5:**
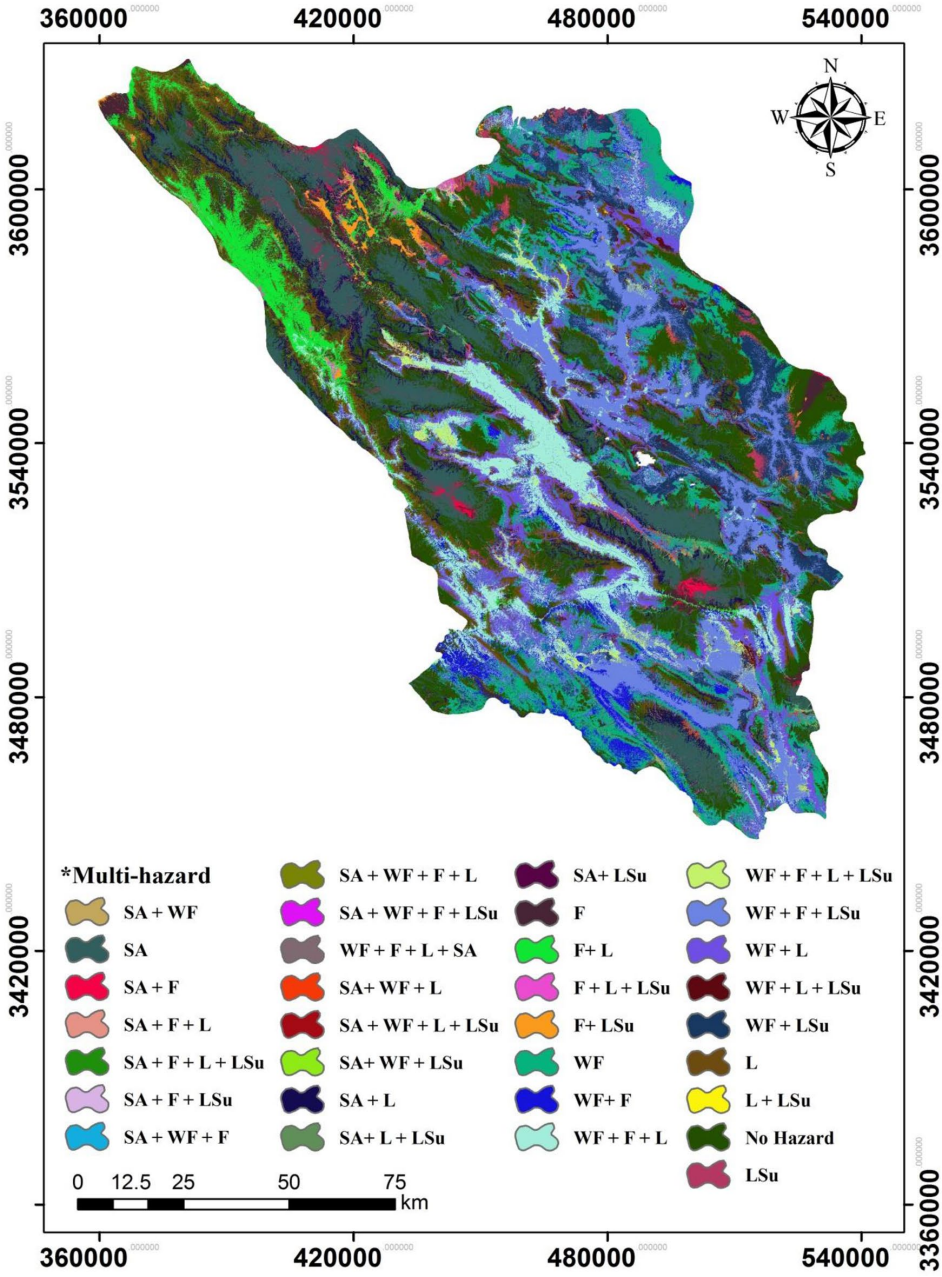
The multi-hazard risk map based on a combination of the five best hazard risk maps for Chaharmahal and Bakhtiari Province (*L Landslide, *LS*u Land subsidence, *F* Flood, *WF* Wildfire, *SA* Snow avalanche).

Snow-avalanche hazard (SAH), landslide hazard (LH), wildfire hazard (WFH), land-subsidence hazard (LSH), and flood hazard (FH) maps were created from the effective factors with the three machine learning models (Fig. 4). First, susceptibility to each hazard was created according to the dependent variables (locations of landslides, floods, avalanches, etc.) and some effective factors (the independent variables) using machine learning techniques. Next, the models with the highest accuracies, determined from ROC-AUC values, were selected and used for multi-hazard mapping. These models were integrated using a Boolean algorithm based on four classes for each hazard—low, moderate, high, and very high. A review of the literature^44,45^ indicated that susceptibility classes of low and moderate were low hazard (0) conditions and high and very high were deemed high hazard (1) conditions. To facilitate integration, the four-class maps produced for each hazard by the best models (from among the three algorithms) were reassigned these two classes: 0 and 1. The maps of the five natural events (flood, landslides, land subsidence, snow avalanches, and wildfires) were combined to create an integrated multi-hazard (MH) map (i.e., MH = SAH + LH + LSH + WFH + FH) in ArcGIS and the result was reclassified (Fig. 5).

#### Accuracy assessment

The accuracy of each of the MH maps was assessed with the training group data (for the goodness-of-fit test) and the validation group data (for the predictive-performance test) using area under the curve (AUC). AUC is a scalar measure that is a threshold-independent method^92,93^. An area of 1 represents perfect classification, while an area of 0.5 or less indicates poor classification of locations by a model^45,94–96^. In the present study, to produce multi-hazard susceptibility maps of snow avalanches, land subsidence, wildfires, landslides, and flood by GLM, FDA, and SVM models a special package was applied in the R software version R 3.5.3. The packages used were "svm"^60,97^, "glm"^55,63^, and "fda"^17,66^.

## Results

### Accuracy assessments of the hazard maps using AUC

Assessing the accuracies of the three machine learning models (Table 2) demonstrated that FDA (for SAH), SVM (LSH), GLM (WH), SVM (LH), and SVM (FH) provided the most accurate models. The values of AUC these five models were all greater than 0.8, indicating strong classification success and confirmed that the models were acceptably accurate.

**Table 2:** AUC values for three machine learning models in mapping natural hazards.

Model	Hazard				
	Flood (F)	Wildfire (WF)	Snow avalanche (SA)	Landslide (L)	Land subsidence (LSu)
SVM	**0.975**	0.835	0.894	**0.841**	**0.943**
FDA	0.962	0.825	**0.912**	0.779	0.920
GLM	0.965	**0.837**	0.909	0.777	0.923

### Integrated multi-hazard (MH) map

The results of the MH map show that the hazards do not overlap (Table 3 and Fig. 5). More than 1/6th (16.51%) of the province is free of all five hazards. Five sixths (83.49%) of Chaharmahal and Bakhtiari Province experiences at least one of the hazards.

**Table 3:** Areas of different classes of various hazards.

Multi-hazard	Area (ha)	Percent (%)
No hazard	269,093.34	16.511
SA	186,049.44	11.416
WF	180,470.97	11.074
WF + L	160,292.88	9.835
WF + F + LSu	136,861.83	8.398
L	133,069.95	8.165
WF + F + L	114,211.8	7.008
WF + LSu	101,536.92	6.230
SA + L	84,893.76	5.209
F + L	65,183.67	4.000
WF + F	55,212.84	3.388
F	45,086.31	2.766
WF + F + L + LSu	22,783.14	1.398
LSu	15,398.73	0.945
WF + L + LSu	12,496.86	0.767
SA + F	12,462.66	0.765
F + LSu	10,308.87	0.633
SA + F + L	7,746.75	0.475
SA + WF + L	6,630.3	0.407
SA + WF	3,918.69	0.240
F + L + LSu	2,751.12	0.169
L + LSu	1,112.31	0.068
SA + WF + F + L	1,084.86	0.067
SA + WF + F	353.25	0.022
SA + F + LSu	273.78	0.017
SA + F + L + LSu	140.67	0.009
SA + LSu	118.53	0.007
SA + WF + F + L	85.77	0.005
SA + L + LSu	48.51	0.003
SA + WF + L + LSu	27.72	0.002
SA + WF + LSu	21.78	0.001

## Discussion

Arid and semi-arid regions of the world experience extreme natural events that threaten the structures and daily functions of localities^98^. Natural hazards can cause a great deal of economic damages^99^, interruptions, injuries, and loss of life. Mountainous regions are among the most disaster-prone parts of the world because of their geological, climatological, and hydrological characteristics^100,101^.

An effective way to begin to manage natural disasters is to map hazards. The information generated can be very useful for effective planning and management of people and activities. Most natural hazards studies have focused on single hazards. Single-hazard approaches focus on hazards as independent phenomena, ignoring the domain of relationships between the hazards^32^ and this may lead to miscalculations of risk^102–107^. A greater emphasis on the interactions between and combinations of hazards’ risks is needed^102^. Studies that have focused on multi-hazard approaches have concluded that there is collectively greater risk from the interactions of multiple hazards than is yielded by simply combining the results of single-hazard studies. The increasing use of GIS in natural resources management and the introduction of various algebraic, statistical, and empirical methods have enabled better assessments of natural hazards. The methods have been developed in different parts of the world based on different conditions and with different amounts of available data, but they have advanced the modeling process and have revealed the spatial distributions of the natural hazards in many study areas.

Several methods have been used to model and map different natural hazards. For example, flood risk has been assessed using support vector machine (SVM), frequency ratio (FR), multivariate statistical analysis, weight of evidence (WoE), analytic hierarchy process (AHP), and decision trees (DTs). The analytic hierarchy process (AHP) method is one of the most common ways to solve problems associated with the use of multiple variables^108,109^ and it is often used in hazard assessments^110^. However, mapping processes are very sensitive to changes in expert’s judgments and to changes in weighting the input variables at the assessment scale and are significant disadvantages^109^. The most popular methods used in landslide risk assessments are neuro-fuzzy inference systems^111^, logistic regression models, analytic hierarchy process, statistical indices^112^, vector based methods^113^, and artificial neural networks^114^. For wildfire risk assessment, probabilistic models and maximum entropy models^115,116^, neural network (NN)^117–119^, fuzzy logic ^120–122^, logistic regression (LR)^21,123–125^, decision tree (DT)^126^, the random forest (RF)^127–129^, and support vector machine (SVM)^24,130^ have been used. Numerous methods have also been used for mapping snow avalanche risk: multi-criteria decision making approaches^131–133^, fuzzy–frequency ratio models^134–136^, numerical methods, dynamic models^137^, and remote sensing-based methods^138,139^. Though remote sensing can provide useful information about snow avalanches, the complex relationships between snow avalanches and geomorphometric variables are often overlooked, and most risk assessments are based on expert opinion. And prediction of land subsidence risk has used methods like artificial neural networks^140^, frequency ratio^141^, logistic regression^142^, and differential radar interferometry^143^.

Machine learning is another modeling technique that is increasingly used to understand the complex relationships between a wide range of independent variables like meteorological factors (winds, air pressure, storm surge, and floods) and a dependent variable^144^. Therefore, these algorithms can aid forecasting of multiple hazards simultaneously, where the environmental conditions vary considerably across a landscape^145^.

In this study, we assessed five hazards in a mountainous region of Iran. To comprehensively assess extreme natural events in the study area, multi-hazard mapping was conducted using three machine learning models. Evaluation of the accuracies of the SVM, GLM and FDA models showed that SVM is most accurate when predicting landslide, land subsidence, and flood risks. GLM is most accurate for wildfire risk. And FDA is most accurate for snow-avalanche risk prediciton (Table 2). The AUCs of the five best models were over 0.8, validating their strong performances^146^ and demonstrating that they (more or less) accurately predicted the patterns of the hazards in the study area. The SVM method also produced very good results for mapping landslide, land subsidence, and flood risks. Li et al.^147^ applied SVM with univariate and multivariate statistical methods to investigate land subsidence. Their results showed that SVM is more accurate than other algorithms they tested. Others have confirmed the high performance of SVM for similar purposes^24,131,148,149^. Studies of landslide risk have also revealed that highly accurate predictions were made with SVM^112,150^. The strong capacity of SVM to predict flood risk has also been demonstrated^151–153^. GLM has been used to predict wildfire risk^123,154–158^. GLMs have proven to acceptably predict wildfire risks in California^155,159^ and Spain^156,160^.

The MH risk map was developed by combining the results produced by the SVM, GLM and FDA approaches. Results demonstrate that using the best machine learning models to predict several hazards yields useful information about their interactions. Multi-hazard relationships are very dependent upon the scale of analysis and the specific sets of hazards. Understanding the relationships and interactions between multiple hazards is an important challenge^103^. This study begins to fill this gap. The results show that all five hazards are absent from 16.5% of the study area. The rest of the study area, 83.5%, is likely to be impacted by at least one of the hazards, however. Pourghasemi et al.^54^ mapped both the individual and collective risks posed by three hazards (floods, forest fires, and landslides) in a multi-hazard study using machine learning techniques. Others have conducted multi-hazard risk assessments, but separately for each risk^46,47,161^.

## Conclusions

As mountainous areas are challenged with a wide array of natural hazards and sites within them are prone to exposures to multiple natural hazards, this study evaluated the spatial distribution of risk from multiple hazards in Chaharmahal and Bakhtiari Province, Iran, using three machine learning models (SVM, GLM and FDA). Identification of high-risk areas is the most important issue for most decision makers and natural resource managers. In this regard, we presented a multi-hazard risk map for five natural hazards (floods, landslides, land subsidence, snow avalanches, and forest fires) in the study area. Evaluation of the accuracies of the maps produced by the SVM, GLM, and FDA models showed that SVM is most accurate model for predicting landslide, land subsidence, and flood risks. GLM is best for wildfire risk prediction. And FDA is best for snow avalanche risk assessment in the region. The results indicate that 16.5% of the study area is not likely to experience any of the five natural hazards, but the rest of province (83.5%) is at risk from exposure to at least one of the five (or several or perhaps all): 11.41% is possesses snow avalanche risk, 11.07% wildfire risk, and 9.83% landslide risk. Each type of machine learning method achieved acceptable levels of accuracy in their predictions. Therefore, these results can be regarded with high confidence and may be used in future studies to examine the spatial distributions of risks from multiple hazards and to provide useful information for proactive management and hazard mitigation.
